# RRH Clustering Using Affinity Propagation Algorithm with Adaptive Thresholding and Greedy Merging in Cloud Radio Access Network

**DOI:** 10.3390/s21020480

**Published:** 2021-01-12

**Authors:** Seju Park, Han-Shin Jo, Cheol Mun, Jong-Gwan Yook

**Affiliations:** 1 Department of Electrical and Electronic Engineering, Yonsei University, Seoul 03722, Korea; dream158@yonsei.ac.kr; 2 Department of Electronics and Control Engineering, Hanbat National University, Daejeon 34158, Korea; 3 Department of Electronic Engineering, Korea National University of Transportation, Chungju 27469, Korea; chmun@ut.ac.kr

**Keywords:** machine learning, clustering, affinity propagation, C-RAN, exterior interference

## Abstract

Affinity propagation (AP) clustering with low complexity and high performance is suitable for radio remote head (RRH) clustering for real-time joint transmission in the cloud radio access network. The existing AP algorithms for joint transmission have the limitation of high computational complexities owing to re-sweeping preferences (diagonal components of the similarity matrix) to determine the optimal number of clusters as system parameters such as network topology. To overcome this limitation, we propose a new approach in which preferences are fixed, where the threshold changes in response to the variations in system parameters. In AP clustering, each diagonal value of a final converged matrix is mapped to the position (x,y coordinates) of a corresponding RRH to form two-dimensional image. Furthermore, an environment-adaptive threshold value is determined by adopting Otsu’s method, which uses the gray-scale histogram of the image to make a statistical decision. Additionally, a simple greedy merging algorithm is proposed to resolve the problem of inter-cluster interference owing to the adjacent RRHs selected as exemplars (cluster centers). For a realistic performance assessment, both grid and uniform network topologies are considered, including exterior interference and various transmitting power levels of an RRH. It is demonstrated that with similar normalized execution times, the proposed algorithm provides better spectral and energy efficiencies than those of the existing algorithms.

## 1. Introduction

The 5th generation mobile communication system (5G system) has three types of requirements: Enhanced mobile broadband, massive machine-type communications, and ultra-reliable and low-latency communications (URLLC) [1]. Among these requirements, reliability is an important factor that directly affects the quality of the 5G system service. Therefore, many studies have focused on this aspect of 5G systems [2,3]. The cloud radio access network (C-RAN) has a centralized processor baseband unit (BBU) and a distributed radio remote head (RRH) to provide a better network structure for URLLC [4]. In the C-RAN of the 5G system, the joint transmission technique between RRHs is an essential technique that can increase reliability by eliminating the overhead caused by the inter-cell interference and hand-off [5,6,7]. There are two types of joint transmission: Non-coherent joint transmission and coherent joint transmission [8,9,10]. As a representative wireless communication technology that uses coherent joint transmission, the cell-free massive multiple-input and multiple-output (MIMO) is capable of simultaneously transmitting high data rates to multiple devices without any interference [11,12,13]. In this study, we assume coherent joint transmission. Although joint transmission exhibits strong performance, the number of RRHs that can be combined on a network is limited. Therefore, it is necessary to appropriately configure a cluster between RRHs based on the channel environment using an appropriate clustering algorithm. Even in traditional wireless sensor networks, clustering algorithms are important for configuring user-oriented multiple sensors. Therefore, many clustering algorithms have already been proposed [14,15,16].

Traditionally, clustering is a method of discovering the regularity present in a large quantity of unstructured information and appropriately grouping this information based on user-defined rules. Depending on how the information is grouped, the interpretation and results can be completely different. Therefore, the user intending to use clustering must adopt an appropriate clustering method to group the desired information. This paper aims to address the clustering problem, which is a non-deterministic polynomial hard problem, using a clustering algorithm with low time complexity and excellent performance. Clustering algorithms are often used in medicine, chemistry, and biotechnology to categorize information [17,18,19]. Among the clustering algorithms, the well-known affinity propagation (AP) clustering algorithm is a representative clustering method based on graph theory [20,21,22,23]. Graph theory-based clustering is characterized by information exchange among data points. This method has a lower time complexity than that of other clustering methods [20]. The AP clustering algorithm is extremely efficient in terms of energy consumption [24]. Furthermore, low time complexity is a key requirement that enables dynamic clustering to be implemented in a wireless communication system where the channel state between a RRH and user equipment (UE) changes in real time.

The AP clustering algorithm is a machine learning-based clustering technique that obtains a reasonable cluster combination and number of clusters through iteration. That is, the number of clusters does not need to be determined before the algorithm is executed. First, a similarity matrix is designed based on the characteristics of the data points, and each component of this matrix is updated and converged through iteration. Finally, the converged matrix is used for clustering [20]. As the similarity matrix is the only input value, the design of the matrix and setting the initial values almost dominate the clustering performance. The similarity matrix is divided into diagonal and off-diagonal components. Diagonal components are called preferences and are terms that pertain mainly to the number of clusters K. Off-diagonal components are values that pertain mainly to the combinations of data points that constitute the cluster. Although it is not an input value of this algorithm, there is one more parameter to effectively control the number of clusters, which is a threshold set to the default value of zero. Among the diagonal components of the final matrix after the iteration is completed, the components that exceed this threshold are called exemplars. The number of exemplars is equal to the number of clusters.

Recently, studies on AP clustering algorithms have focused on improving the joint transmission performance among RRHs in wireless communication networks. In these studies, the similarity matrix is designed on the basis of the expected or instant value of the channel gain between transmitting and receiving nodes, because the channel gain is a representative index that measures the similarity between wireless communication nodes (data points) [25,26,27]. Wesemann el al. [25] verified that the application of a similarity metric, defined as the sum of mutual inter-cell interference, can outperform static clustering in uplink joint transmission. Zhang et al. [26] proposed BS clustering using a similarity matrix consisting of the signal-to-interference and noise ratio (SINR) gain of joint transmission over non-joint transmission in a downlink pico-cellular network. Both the offline and online phases of AP clustering are also provided for practical implementation. This approach to clustering achieved better performance than that of the existing clustering algorithms. In a typical small-cell C-RAN, the RRHs are not sufficiently separated out of the reach of radio waves, causing exterior interference [28], which refers to interference emitted from RRHs connected to the adjacent BBUs using the same frequency and resource elements. With this background, Park et al. [27] proposed a new preference for controlling the number of clusters considering not only inter-cluster and intra-cluster interferences but also exterior interference.

### Contribution and Organization

As all AP clustering methods proposed in previous studies [25,26,27] use a threshold fixed to zero, the change in the number of clusters is significant in response to a change in the preference value. Therefore, the clustering performance also varies significantly. Studies have suggested that the number of clusters increases as the value of the preference increases [21]. However, there is no formula that specifically defines the relationship between the two values. Therefore, the preference value must be swept to achieve optimal clustering performance. However, this approach inevitably entails an extremely high computational complexity, as shown in [29].

To overcome this problem, we propose a new approach in which the preference value is fixed and the conventional fixed zero threshold is replaced with a value that changes adaptively in response to various system parameters. To determine the adaptive threshold, the diagonal components of the converged responsibility matrix R + availability matrix A (R+A matrix) that has finished iteration in the similarity matrix (see Section 3 Algorithm 1 for detail) are imaged in three dimensions as the RRH position. The Otsu method, which determines the threshold that divides the background and foreground based on statistical decision theory using a gray-scale histogram, is applied to the imaged information. The proposed AP clustering technique using the Otsu threshold works effectively in situations where many exemplars are determined based on a fixed preference and adjusts the number of exemplars reasonably [30]. Since an exemplar represents the center of a cluster, the adjacent exemplars indicate that the interference among clusters (inter-cluster interference) can be enlarged. The existing AP clustering method does not have the ability to correct the exemplars adjacent phenomenon. The greedy merging algorithm can increase the performance of the AP clustering system by combining the exemplars into one when they are adjacent to one another and solve this problem. We verified that the proposed AP clustering algorithms have better performances than those of the existing AP clustering algorithms using the Otsu threshold and greedy merging methods, which are simpler than the existing preference sweeping AP clustering algorithms. In Section 3, the process flow of the AP clustering algorithm is discussed in detail.

**Figure 1:**
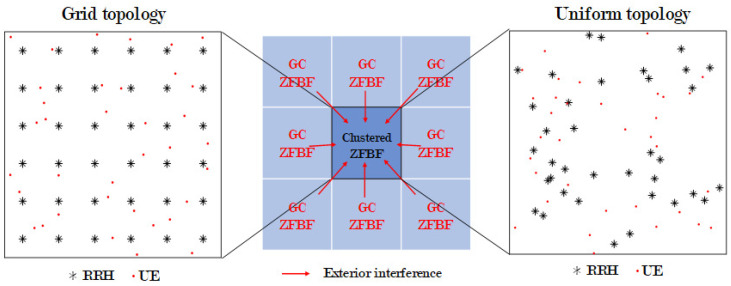
Concept of exterior interference from eight baseband units (BBU) environment with globally coordinated zero-forcing beamforming (ZFBF) in two topologies.

In this study, the conventional and proposed AP clustering algorithms are compared using a system-level simulation in a small-cell C-RAN with various realistic models and parameters. The simulation considers not only inter-cluster and intra-cluster interferences but also exterior interference, and a channel model using stochastic path loss and shadowing for mmWave of IEEE TR 38.900 and flat fading [31], as well as various levels of RRH transmitting power in exterior areas. Moreover, the performance evaluation is performed for both the grid and uniform random network topology, as depicted in Figure 1.

This paper is organized as follows. Section 2 describes the system model and simulation settings. The conventional and proposed AP algorithms are discussed in Section 3. Performance evaluation and simulation results are presented in Section 4, and concluding remarks summarize the main results in Section 5.

## 2. System Model

**Figure 2:**
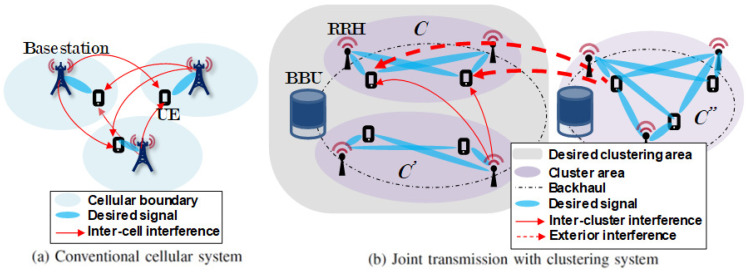
Concept of conventional cellular and joint transmission a with clustering system. (**a**): conventional cellular system; (**b**): joint transmission with clustering system.

In this section, the spectral efficiency (SE) for performance verification is derived, and the environment for the system-level simulation is defined. Figure 1 depicts the total area of the system-level simulation. This area is divided into nine sections in two network topologies. Each of these nine sections has 36 RRHs and 36 UE connected to one BBU. The scheduling algorithm is generally not assumed. If the scheduling algorithm is assumed, it is possible to simulate a situation in which the number of UE is significantly larger than that of RRHs. The clustered ZFBF area in Figure 1 is depicted in Figure 2b as the desired clustering area. This is the area where the performance of the existing clustering algorithm is compared with that of the algorithm proposed in this paper. This area is evaluated using two network topologies: Grid and uniform. In the first topology, the RRHs are installed in a grid pattern in the form of a square cell, with the distance between adjacent RRHs being equal. In this topology, the UE are randomly located within the square cell boundary of the central RRH. In the second topology, the RRHs and UE are randomly located within the area. In this topology, the RRH with the highest reception power at the UE is assumed to be the RRH serving the UE. The remaining eight areas in Figure 1 are regions for exterior interference. These areas use the same frequency and resource blocks as does the middle area and assume global coordination ZFBF i.e., K=1. The following describes the evaluation of the clustering algorithm in the middle area. Figure 2a depicts the desired signal in a conventional cellular system with inter-cell interference, and Figure 2b depicts the desired cluster region *C* receiving inter-cluster interference from $C^{\prime }$ and exterior interference from $C^{\prime \prime }$. The received signal at user *i* served by the desired cluster *C* with ZFBF can be described as:

(1) $$y_{i}=\underset{\mathrm{Desired}\mathrm{signal}}{\underset{︸}{h_{i}^{C}w_{i}^{C}s_{i}}}+\underset{\mathrm{Intra}-\mathrm{cluster}\mathrm{interference}}{\underset{︸}{\sum_{j=1,j\neq i}^{N}h_{i}^{C}w_{j}^{C}s_{j}}}+\underset{\mathrm{Inter}-\mathrm{cluster}\mathrm{interference}}{\underset{︸}{\sum_{k=1,k\neq i}^{M}\sum_{l\in C^{\prime }}h_{i_{k}}^{C^{\prime }}w_{l_{k}}^{C^{\prime }}s_{l_{k}}}}+\underset{\mathrm{Exterior}\mathrm{interference}}{\underset{︸}{\sum_{o=1}^{E}\sum_{q\in C^{\prime \prime }}h_{i_{o}}^{C^{\prime \prime }}w_{q_{o}}^{C^{\prime \prime }}s_{q_{o}}}}+n_{i},$$

where $s_{i}$ is the desired signal from the desired *i*th RRH to the desired *i*th UE, and $h_{i}^{C}$ is the channel gain received in the desired cluster *C*. *N* is the number of RRHs in desired cluster *C*. $s_{j}$ is the signal from the *N* RRHs, except for the *i*th RRH in cluster *C* to the *i*th UE. Assuming perfect channel state information, the intra-cluster interference converges to zero owing to the orthogonal characteristics of the ZF precoding. As depicted in Figure 1 and Figure 2b, $h_{i_{k}}^{C^{\prime }}$ is the channel gain received from the *M* RRHs of other cluster $C^{\prime }$ in the desired area, and $s_{l_{k}}$ is the signal from the *M* RRHs of the other cluster $C^{\prime }$ to the *i*th UE. $mathbfh_{i_{o}}^{C^{\prime \prime }}$ is the channel gain received from the exterior clusters $C^{\prime \prime }$ in the eight exterior areas (E=8). $n_{i}$ denotes the additive white Gaussian noise with zero mean and variance $\sigma_{i}^{2}$ of the *i*th UE. The channel coefficient h used in (1) assumes a flat fading channel. For the path-loss and shadowing model, the urban micro-street canyon model of IEEE TR 38.900 is used [31]. $s_{i}$, $s_{j}$, and $s_{l_{k}}$ occupy the same resource block. In (1), interference is categorized into three types: Intra-cluster, inter-cluster, and exterior interferences. The intra-cluster interference is generated inside the precoding matrix of the desired cluster *C*, inter-cluster interference is generated by the weight matrices from the other clusters $C^{\prime }$ in the desired area, and exterior interference is applied from clusters $C^{\prime \prime }$ of the eight exterior areas. w is the normalized ZF weight vector for each channel coefficient vector, h, and is given by:

(2) $$\tilde{W}=\left[{\tilde{w}}_{1},{\tilde{w}}_{2},...,{\tilde{w}}_{Q}\right]=H^{H}{\left(HH^{H}\right)}^{-1}$$

$$w_{i}={\tilde{w}}_{i}/\left|\right|{\tilde{w}}_{i}\left|\right|,$$

where *H* is the Hermitian of the matrix, and *Q* is the number of UE constituting the desired cluster. The SINR of the *i*th user in the downlink are given by:

(3) $${\mathrm{SINR}}_{i}=\frac{P_{i}{\left|h_{i}^{C}w_{i}^{C}\right|}^{2}}{\underset{\mathrm{Inter}\mathrm{interference}}{\underset{︸}{\sum_{k=1,k\neq i}^{M}\sum_{l\in C^{\prime }}P_{i}{\left|h_{i_{k}}^{C^{\prime }}w_{l_{k}}^{C^{\prime }}\right|}^{2}}}+\underset{\mathrm{Exterior}\mathrm{interference}}{\underset{︸}{\sum_{o=1}^{E}\sum_{q\in C^{\prime \prime }}P_{e}{\left|h_{i_{o}}^{C^{\prime \prime }}w_{q_{o}}^{C^{\prime \prime }}\right|}^{2}}}\;+\sigma_{i}^{2}},$$

where the intracluster interference is eliminated by ZF precoding. $\sigma_{i}^{2}$ denotes the additive white Gaussian noise with zero mean and variance $\sigma_{i}^{2}$. $P_{e}$ is the transmitting power of the RRH in the eight exterior areas (E=8), and $P_{i}$ is the transmitting power of the RRHs in the desired clustering areas *C* and $C^{\prime }$. This study evaluates the SE performance by fixing $P_{i}$ and changing only the $P_{e}$ of the RRHs in the exterior area $C^{\prime \prime }$. The SE of the *i*th UE is obtained from the SINR as follows:

(4) $${\mathrm{SE}}_{i}=\log_{2}\left(1+{\mathrm{SINR}}_{i}\right).$$

In this paper, we compared and evaluated the joint transmission performance of the RRH group by applying the clustering algorithm using (4). In addition, the energy efficiency performance was evaluated as shown in (5):

(5) $${\mathrm{EE}}_{i}=\frac{{\mathrm{SE}}_{i}}{P_{i}}.$$

## 3. RRH Clustering with AP Algorithm Schemes

This section describes an AP clustering algorithm that determines a joint transmission group among RRHs in a small-cell C-RAN environment. The AP clustering algorithm is a graph theory-based clustering method that uses information exchange among data points [20]. Parametric-based clustering has a disadvantage in that the clustering performance variance is large because the cluster configuration can vary according to the initial value. However, the AP clustering method always creates the same cluster when the initial value is set, but it has the disadvantage of the time complexity being slightly higher than the parametric-based method because of the iteration process. The AP clustering method is particularly suitable for joint transmission systems in wireless communication networks. The AP algorithm has an input value based on initial information among data points called the similarity matrix S. This matrix contains information relating data point pairs, so it is a square matrix with the size of the number of data points, and is divided into off-diagonal components and diagonal components called preferences. These two components play different roles in creating a cluster. Off-diagonal components are values related to a set of data points in a cluster. Diagonal components are values related to *K*. Therefore, both of these two components have a very important effect on the cluster performance. However, the focus of this study is on improving the clustered joint transmission performance by appropriately adjusting *K*:

(6) $$s\left(i,p\right)=\left\{\begin{array}{cc} \log(\frac{SINR_{CoMP}\left(i,p\right)}{SINR_{non}^{i}}), & i\neq p\\ \varepsilon \cdot [\log\left(\frac{1}{SINR_{non}^{i}}\right)], & i=p \end{array}\right.$$

**Figure 3:**
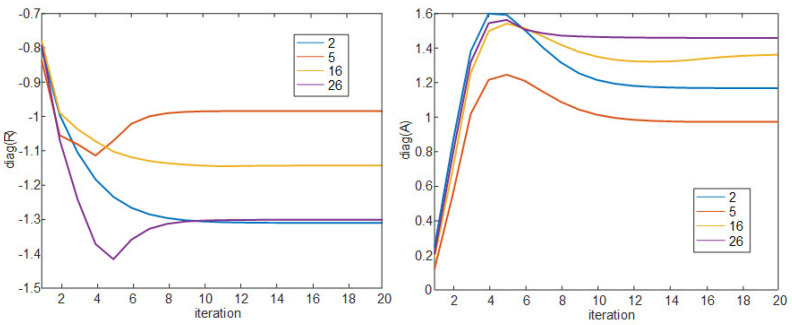
Diagonal component values of R,A matrix by iteration in Algorithm 1 (2, 5, 16, and 26 data points).

**Figure 4:**
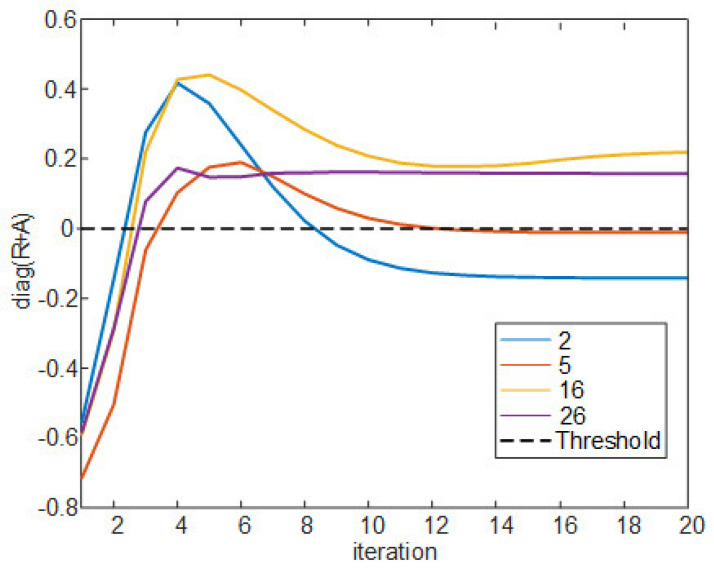
Diagonal component values of R+A matrix by iteration in Algorithm 1 (2, 5, 16, and 26 data points) with 0 threshold.

As shown in Algorithm 1, the similarity matrix S is the input value, and the responsibility matrix R and the availability matrix A, which have the same matrix size as S, are set to 0 matrix. Lines 3 to 7 calculate and update the R and A matrices. ψ and ζ in line 8 are oscillating factors, representing the weights for the past and present values of the R and A matrix that are iterated by lines 9 to 13, and both values are defined as 0.5. Lines 14 to 20 determine the exemplar. Among the diagonal components of the R+A matrix after iteration, components larger than threshold 0 are called the exemplar. The cluster set included in the exemplars is determined by line 19. Figure 3 is a graph showing the convergence process of the diagonal components of matrices R and A with the iteration progresses for data points 2, 5, 16, and 26 as an example. In the case of Algorithm 1, it can be observed that convergence is usually achieved with 10 iterations. Figure 4 shows the convergence process of the diagonal components of the R+A matrix, and the two data points 16 and 26 selected as the exemplar larger than threshold 0. (5) is the similarity matrix used in the uplink joint transmission proposed in [26]. In this paper, this similarity matrix is used for down-link joint transmission. The off-diagonal components of this similarity matrix are defined as the value dividing the down-link SINR to the *i*th UE by the SINR value when non-CoMP pair CoMP between the *i*th RRH and all other RRHs (*p* indexing). This is the value representing the gain when performing joint transmission with the *i*th RRH. Here, the meaning of $SINR_{non}^{i}$ indicates the ratio of the sum of the interference signal power received from all RRHs except the *i*th RRH plus noise power and the desired signal power received from the *i*th UE. The meaning of $SINR_{CoMP}\left(i,p\right)$ indicates the ratio of the desired signal power received by the *i*th UE from the joint transmission of *i*th RRH and *p*th RRH and the sum of the interference signal power received from all RRHs except ith RRH and pth RRH plus noise power. Diagonal components can be obtained by multiplying the values remaining after excluding the pair CoMP SINR from the off-diagonal components by ε, a coordinative parameter. In (5), ε adjusts the size of the preference and this value is determined to be 0.3. As ε decreases, the value of the diagonal components decreases and *K* decreases, and as ε increases, *K* also increases. We implement a basic AP algorithm using this similarity matrix in the following subsections, and propose a new clustering algorithm by adding an image processing method and another clustering algorithm.
**Algorithm** **1** AP clustering algorithm1:(A) Initialization: Similarity matrix S according to (5)2:Set initial availability matrix A=[0], responsibility matrix R=[0], and # of cluster K=03:Calculate R(i,:) and A(:,p)4:$r\left(i,p\right)=s\left(i,p\right)-\max_{p^{\prime }\neq p}\left(a\left(i,p^{\prime }\right)+s\left(i,p^{\prime }\right)\right)$5:$a\left(i,p\right)=\min\left(0,r\left(p,p\right)+\sum_{i^{\prime }\notin i,p}\max\left(0,r(i^{\prime },p)\right)\right)$6:$a\left(p,p\right)=\sum_{i^{\prime }\neq p}\max\left\{0,r\left(i^{\prime },p\right)\right\}$7:Update R(i,:) and A(:,p)8:Oscillatory decay: (φandζ∈[0,1])9:(B) Iteration:10:**while** Convergence **do**11:      R(iter)=φ·R(iter)+(1−φ)·R(iter−1)12:      A(iter)=ζ·A(iter)+(1−ζ)·A(iter−1)13:(C) Exemplar judgment:14:**for**ind=1 to number of RRH **do**15:      **if** r(ind,ind)+a(ind,ind)>0 **then**16:            K = K + 117:      exemplar$\left(ind\right)={\mathrm{argmax}}_{p}$$\left(a(\mathrm{ind},p)+r(\mathrm{ind},p)\right)$18:(D) Output: exemplar, set of clusters, and K

### 3.1. Proposed Clustering Algorithm 1

**Figure 5:**
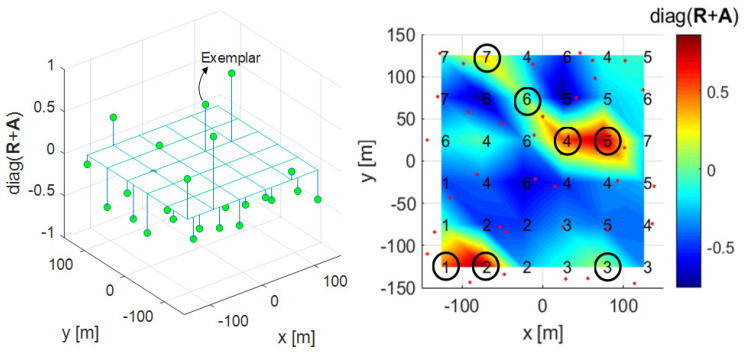
3-D plot (left) and mesh plot (right) with the diagonal components of the R+A in Algorithm 1.

**Figure 6:**
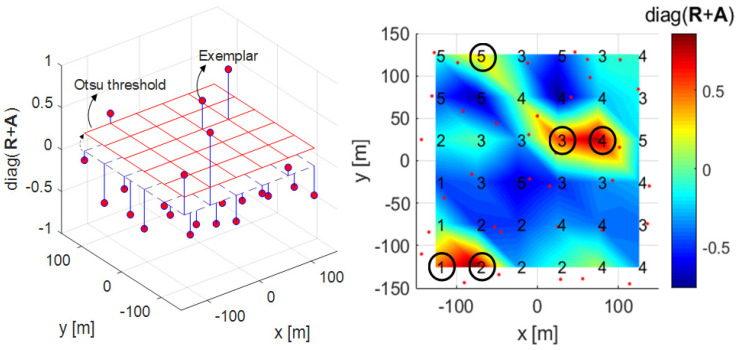
3-D plot (left) and mesh plot (right) with Otsu threshold applied to the diagonal components of R+A in Algorithm 2.

**Figure 7:**
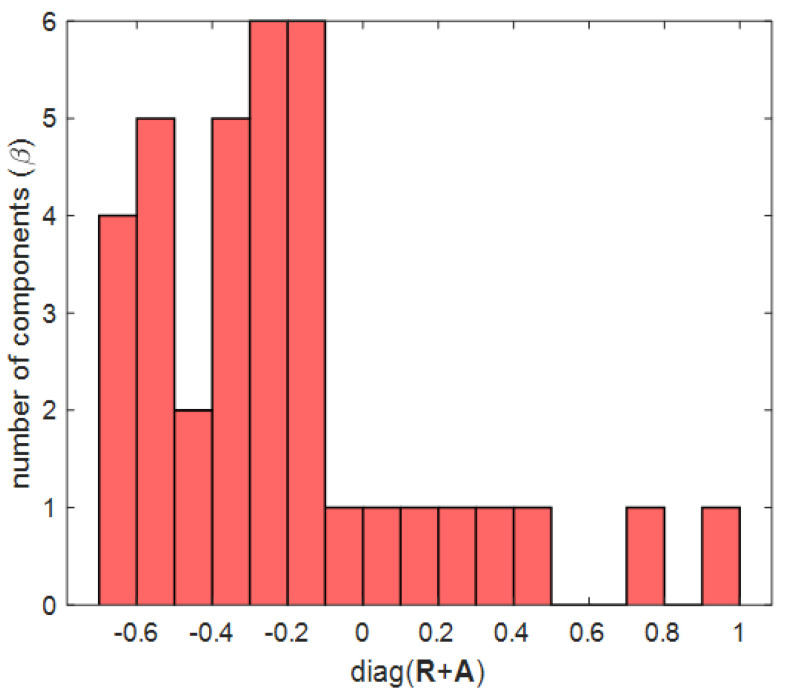
Histogram of diagonal component values of R,A matrix for Otsu threshold.

**Figure 8:**
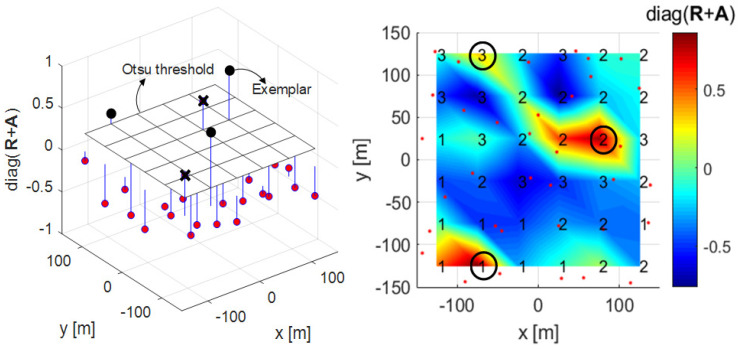
3-D plot (left) and mesh plot (right) with Otsu threshold and greedy merging algorithm applied to the diagonal components of R+A in Algorithm 3.

The proposed clustering Algorithm 1 is a method of changing the fixed threshold 0 of the conventional AP clustering algorithm to a more suitable value for a given environment. One of the traditional image processing methods, Otsu’s method, is used in this clustering algorithm [30]. Otsu’s method can be selected as a method for binarization and transforms an image with several levels of gray including white and black into an image with only white and black. In [30], the criterion for dividing white and black is called the Otsu threshold. In this study, as a result of the mesh on the right of Figure 5, Figure 6, Figure 7 and Figure 8, the diagonal components of the R+A matrix that determine the cluster in AP clustering are imaged through mapping to the topology. In Algorithm 2, the diagonal components of the R+A matrix mapping information are used to determine the Otsu threshold in the Otsu’s method. The process of determining the Otsu threshold uses the distribution of black and white in the gray-scale histogram, and can be explained by (6)–(9). In the example of Figure 5, Figure 6, Figure 7 and Figure 8, the diagonal components of the R+A matrix can be represented as a gray-scale histogram as shown in Figure 7. (6) shows the foreground and background weight value of the gray-scale histogram, and shows black as foreground(*f*) and white as background(*b*). *T* is the bin index, $T_{f}+T_{b}=A$, and *A* is the total number of bins. For both $T_{f}$ and $T_{b}$, all values from 0 to *A* are obtained for (6)–(9) (see Figure 7 as an example, A=17). (7) shows the foreground and background mean value of the gray-scale histogram, and γ shows the middle value of the bin width (Figure 7 as an example, −0.65, −0.55, …, 0.95). (8) shows the foreground and background variance value of the gray-scale histogram. (9) indicates within class variance, and eventually finds the index *T* that minimizes $\sigma_{wcv}^{2}$, and determines $\gamma_{T}$ with that index as the Otsu threshold.

(7) $$W_{T_{f,b}}=\frac{\sum_{\alpha =0}^{T_{f,b}}\beta_{\alpha }}{\mathrm{tr}(R+A)}$$

(8) $$\mu_{T_{f,b}}=\frac{\sum_{\alpha =0}^{T_{f,b}}\left(\gamma_{\alpha }\beta_{\alpha }\right)}{\sum_{\alpha =0}^{T_{f,b}}\beta_{\alpha }}$$

(9) $$\sigma_{T_{f,b}}^{2}=\frac{\sum_{\alpha =0}^{T_{f,b}}\left[{\left(\gamma_{\alpha }-\mu_{T_{f,b}}\right)}^{2}\beta_{\alpha }\right]}{\sum_{\alpha =0}^{T_{f,b}}\beta_{\alpha }}$$

(10) $$\sigma_{wcv}^{2}=W_{T_{b}}\sigma_{T_{b}}^{2}+W_{T_{f}}\sigma_{T_{f}}^{2}$$

**Algorithm** **2** Proposed clustering Algorithm 11:(C) Exemplar judgment:2:**for**ind=1 to number of RRH **do**3:      **if** r(ind,ind)+a(ind,ind)> Otsu threshold **then**4:          K = K + 15:       exemplar$\left(ind\right)={\mathrm{argmax}}_{p}$$\left(a(\mathrm{ind},p)+r(\mathrm{ind},p)\right)$6:(D) Output: exemplar, set of clusters, and K

The figure on the right of Figure 5 shows the diagonal components of the R+A matrix as a mesh plot in the desired clustering area *C* and C’ in Figure 1. The left figure of Figure 5 shows the diagonal components of the R+A matrix and threshold 0 at each RRH position as a 3-D plot. In these two figures, the diagonal component values of the R+A matrix having a threshold of 0 or more become exemplars, and in this case, there are 7 exemplars (K=7). Algorithm 2 is the proposed clustering Algorithm 1 to determine the Otsu threshold by constructing a grayscale histogram of the diagonal component values of each RRH’s R+A matrix. The left figure of Figure 6 shows that the threshold is changed by Otsu’s method, and the number of exemplars is eventually changed from 7 to 5. In the figure on the right of Figure 5, exemplars 2 and 7 have lower values than the Otsu threshold, and therefore these data points cannot be selected as exemplars in the figure on the right of Figure 6. Algorithm 2 represents the proposed clustering Algorithm 1, lines 1 to 13 are the same as for Algorithm 1, and there is a difference in how the exemplar is determined after that.

### 3.2. Proposed Clustering Algorithm 2

The second proposed clustering algorithm is a clustering technique that uses a greedy merging algorithm in addition to the proposed clustering Algorithm 1 proposed in Section 3.1. In this paper, a greedy merging algorithm creates one representative exemplar by grouping adjacent exemplars if exemplars are adjacent to each other. An exemplar serves as the center of the cluster, and there will be greater interference when these exemplars are adjacent to each other. Algorithm 3 shows the process flow of the proposed clustering Algorithm 2. Lines 1 to 13 are the same as for Algorithm 1, and lines 14 to 18, the process of applying the Otsu threshold, are the same as for Algorithm and these lines apply the Otsu threshold. Lines 19 to 23 determine the exemplar with the largest diagonal component of R+A as the representative exemplar when it finds an adjacent exemplar among the exemplars that exceed the Otsu threshold. In Figure 6, 2, 3, and 4 exemplars are adjacent among the 5 exemplars that have passed the Otsu threshold. As exemplar 3 has the highest value, the rest are eliminated as exemplars, and exemplar 3 becomes the representative.
**Algorithm** **3** Proposed clustering Algorithm 21:(C) Exemplar judgment:2:**for**ind=1 to number of RRH **do**3:      **if** r(ind,ind)+a(ind,ind)> Otsu threshold **then**4:            K=K+15:      **if** A exemplar has adjacent exemplar **then**6:            K=K − number of adjacent exemplar7:      **else** Overlapped adjacent exemplar8:            K=K9:        exemplar$\left(ind\right)={\mathrm{argmax}}_{p}$a(ind,p)+r(ind,p)10:(D) Output: exemplar, set of clusters, and K

### 3.3. Maximum Performance with AP Algorithm

This method selects the most suitable *K* from a given similarity matrix and serves as the upper bound of the three AP-based clustering algorithms. The method of selecting the exemplar in Algorithm 4 is to calculate (3) in all cases from when *K* is 1 to the number of RRHs (=36), and the exemplar is the *K* having the highest SINR. This clustering method provides the optimal performance for *K*. Therefore, in the Section 4, this method will be referred to as AP w/max *K*, and the results of this algorithm will also be derived to address the extent to which the proposed clustering algorithms can exhibit a performance comparable to that of this clustering algorithm.
**Algorithm** **4** Maximum performance with AP algorithm1:(C) Exemplar judgment:2:**for**t=1 to number of RRH **do**3:      K=K+t4:      temporal exemplar(1 to $t)={\mathrm{argmax}}_{p}$a(t,p)+r(t,p)5:      Solve the (3) - SINR6:      Find K←max(SINR)7:(D) Output: exemplar, set of clusters, and *K*

## 4. Simulation Results

In this section, we compare the SE performance against the γ of several clustering methods in grid and uniform topologies with the C-RAN environment designed in Section 2 and compare the normalized execution time. The following shows the six different clustering methods covered in this section:

1. **AP w/ max *K***: AP clustering algorithm with max *K* exhibits the best performance for Algorithm 1 and is shown in Algorithm 4;
2. **Proposed AP 2**: AP clustering algorithm with ghd Otsu threshold and the greedy merging algorithm (Algorithm 3);
3. **Proposed AP 1**: AP clustering algorithm with Otsu threshold (Algorithm 2);
4. **Conventional AP 1** [26]: Conventional AP clustering Algorithm 1;
5. **Conventional AP 2** [25]: Conventional AP clustering Algorithm 2;
6. **Static CoMP**: Static CoMP scheme assumes coordination of four adjacent cells (K=9).

**Figure 9:**
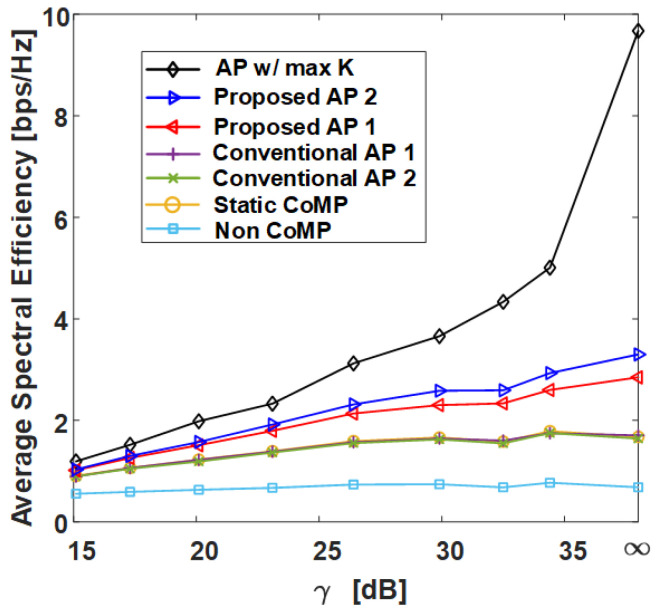
Average spectral efficiency of various clustering schemes with respect to γ in grid topology. AP: Affinity propagation.

**Figure 10:**
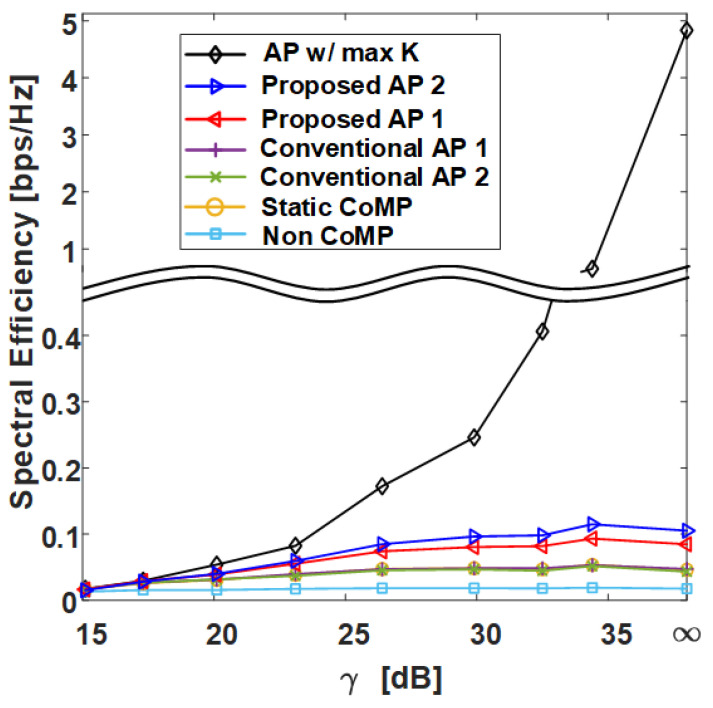
Spectral efficiency at 5% outage level of various clustering schemes with respect to γ in grid topology.

**Figure 11:**
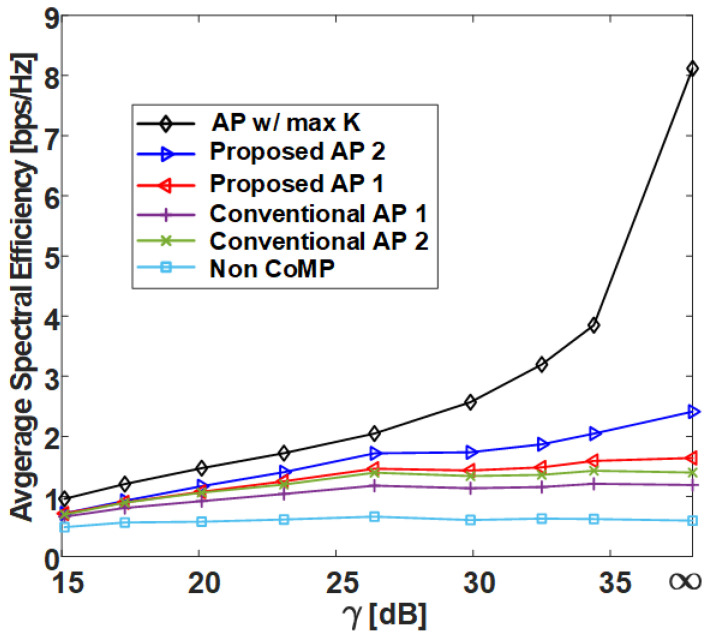
Average spectral efficiency of various clustering schemes with respect to γ in uniform topology.

**Figure 12:**
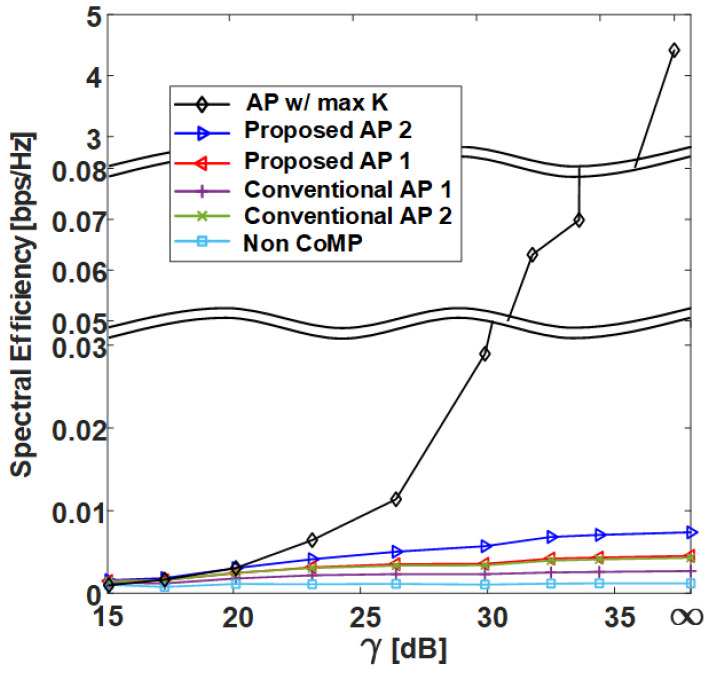
Spectral efficiency at 5% outage level of various clustering schemes with respect to γ in uniform topology.

However, in the uniform topology, the static CoMP method is difficult to define, and therefore it is not added to the result. The non-CoMP result is also added as the lower bound result. Table 1 shows the simulation parameters used in the system-level simulator to derive the results. $P_{i}$, which has a direct effect on the γ value, is set to 25.4 dBm, and $P_{e}$ is set to increase by 3 dBm from 10 dBm to 31 dBm, where $\gamma =E\left(I_{Non-CoMP}\right)/E\left(I_{exterior}\right)$ [dB] on the x-axis is defined as the ratio of the average non-CoMP interference power to the average exterior interference power. Thus, the γ=∞ values in Figure 9, Figure 10, Figure 11 and Figure 12 indicate that the exterior interference is 0. The number of RRHs and UEs is set to 36, and each RRH and UE has a single antenna. In the grid topology, the inter-site distance between RRHs is assumed to be 50 m. In the uniform topology, the inter-site distance is not defined, but the RRH has a random location within the same area as the grid topology. The clustering SE performance is calculated by (4) in the situation where $P_{i}$ and $P_{e}$ are set, and both average SE and 5-percentile UE SE results are derived from Figure 9, Figure 10, Figure 11 and Figure 12.

Figure 9 and Figure 11 show the average SE by γ in grid topology and uniform topology, respectively, and show that the proposed clustering Algorithms 1 and 2 exhibit better performance than the conventional clustering algorithms. The proposed clustering Algorithm 2 shows better performance than the proposed clustering Algorithm 1. Figure 10 and Figure 12 show the results of 5-percentile UE SE in the grid topology and uniform topology, respectively. Figure 10 and Figure 12 show the results of 5-percentile UE SE in grid topology and uniform topology, respectively. The trend is similar to the average SE result, but the SE performance of **AP w/ max *K*** is much better than that of the other clustering algorithms. The reason is that the proposed clustering Algorithms 1 and 2 are clustering methods that consider only *K* without considering the combination of clusters.

**Figure 13:**
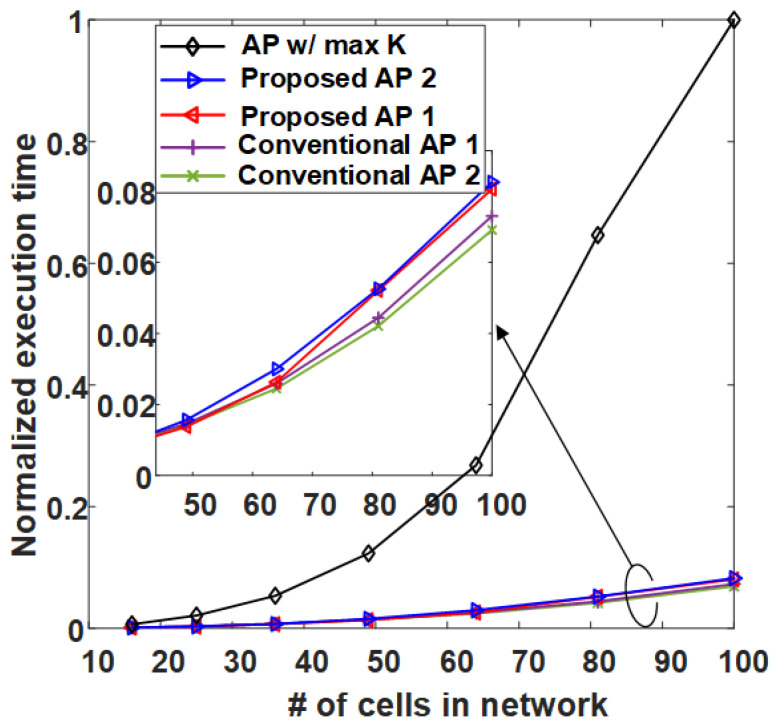
Average normalized execution time comparison of the conventional and the proposed clustering algorithms.

**Figure 14:**
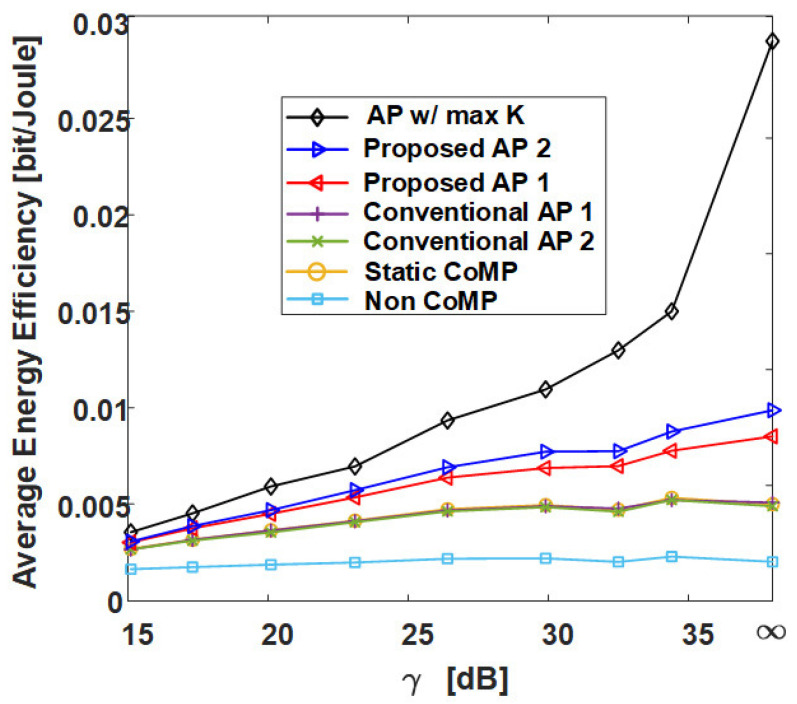
Average energy efficiency of various clustering schemes with respect to γ in grid topology.

**Figure 15:**
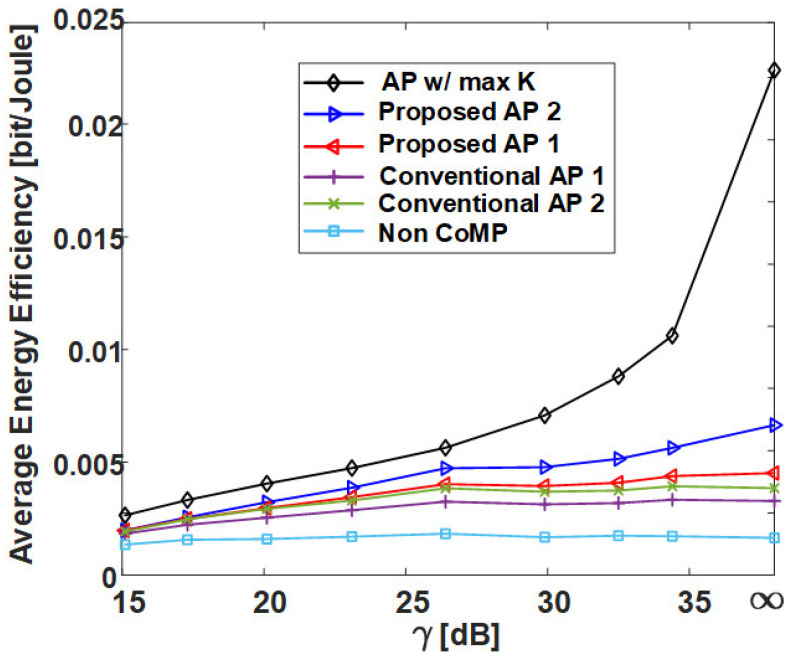
Average energy efficiency of various clustering schemes with respect to γ in uniform topology.

Figure 13 is the result of calculating the normalized execution time of AP-based clustering methods by the number of cells (RRHs) in the network. In Figure 13, the conventional AP clustering Algorithms 1 and 2 do not search the preference but use a initially defined similarity matrix. Naturally, the result of **AP w/ max *K*** shows the highest normalized execution time because the iteration is performed at least 36 times (the number of RRHs) more than the other algorithms. However, although the proposed clustering algorithms exhibit high performance in Figure 9, Figure 10, Figure 11 and Figure 12, they have a normalized execution time similar to that of the other existing AP clustering algorithms. Hence, the proposed clustering algorithms exhibit better performance than the conventional clustering algorithms without a significant increase in time complexity. This result is excellent in terms of energy efficiency because it shows a similar time complexity even with a better clustering performance than the conventional clustering algorithm. Figure 14 and Figure 15 show the average energy efficiency according to (5). This results show that the proposed AP clustering algorithm has a higher performance than the existing AP clustering algorithm in terms of energy efficiency.

## 5. Conclusions

We proposed the use of adaptive thresholding and greedy merging for the AP clustering of the RRHs for dynamic joint transmission. The proposed method is a simple imaging method, without preference searching and the accompanying high time complexity. The clustering performance for a varying γ value, which is the ratio of the exterior interference to the non-CoMP interference, was analyzed in grid and uniform network topologies. While the existing AP-based clustering algorithms create an excessive number of clusters for some channel environments, the proposed clustering Algorithms 1 and 2 generated a relatively lower number of clusters, providing up to 37.7% and 102.4% improved SEs, respectively. Furthermore, the increases in the normalized execution time were small, at most 10.4% and 13%, respectively. Moreover, similar trends for SE were indicated for both the grid and uniform topologies. In addition, we could infer the average SE result of the actual distribution of RRHs as a value between the grid and uniform topology results [32]. The proposed algorithms could be extended to consider a more realistic joint transmission, such as imperfect symbol synchronization owing to the time difference of arrival, in future studies. Furthermore, we intend to perform an additional experimental verification of the results of this study.

## Figures and Tables

**Table 1:** Simulation parameter. RRD: Radio remote head.

Parameter	Value
Center frequency (GHz)	28
Bandwidth (MHz)	20
Number of RRHs and UEs	36
MS receive antenna	1
RRH transmit antenna	1
$P_{i}$ (dBm)	25.4
$P_{e}$ (dBm)	10,13,16,19,22,25,28,31
RRH inter-site distance (m)	50
Height of RRH (m)	5
Height of MS (m)	1.5

## Data Availability

Not applicable.
